# Stem cell-derived exosomes in tissue regeneration of oral and maxillofacial region: A systematic review

**DOI:** 10.1097/MD.0000000000046948

**Published:** 2026-01-02

**Authors:** Qianlei Dai, Qianfen Dai

**Affiliations:** a Department of Stomatology, Liupanshui Dais Dental Hospital, Liupanshui, Guizhou Province, China.

**Keywords:** exosomes, oral and maxillofacial region, periodontal regeneration, stem cells

## Abstract

**Background:**

Oral and maxillofacial diseases are often accompanied by tissue defects and damages, which are difficult to repair and affect patients both physically and psychologically as well as daily life. Recent 5 years studies have proved that stem cell-derived exosomes possess a broad clinical application in regenerative dentistry by promoting hard and soft tissue regeneration, angiogenesis, nerve repair, and wound healing, inhibiting inflammation and apoptosis, and modulating immune responses. The purpose of this review is to sum up the current state of research on stem cell-derived exosomes in periodontal regeneration and to discuss the current challenges and future directions.

**Methods:**

This review article was conducted using electronic databases such as PubMed and Web of Science. The search included studies published up until January 1, 2025.

**Results:**

Stem cell-derived exosomes are a kind of membrane vesicles that encapsulate proteins, RNAs, and other substances, which are one of the important strategies for tissue regeneration and repair. Stem cell-derived exosomes play an important role in the field of periodontal regeneration, including dental pulp, periodontium, jawbone, temporomandibular joint, maxillofacial soft tissue, and nerve. Mechanistically, stem cell-derived exosomes alleviate oral and maxillofacial diseases by promoting tissue regeneration, osteogenesis, odontogenesis, angiogenesis, axonal regeneration, and suppressing inflammation.

**Conclusion:**

Stem cell-derived exosomes may serve as a potential therapeutic strategy for oral and maxillofacial diseases.

## 1. Introduction

In oral and maxillofacial region, bone or cartilage defects caused by trauma, malignancy, and congenital anomalies can lead to both functional impairments and cosmetic issues.^[1]^ Meanwhile, tumor operations,^[2]^ orthognathic procedures,^[3]^ and 3rd molar extractions^[4]^ can also result in neurological disorders in the oral and maxillofacial region. To repair irreversible skeletal damage or recover lost functions, reconstructive surgeries for bone defects and nerve injuries are the current gold standard choice.^[5,6]^ Currently, autografts are an effective therapeutic strategy to repair oral and maxillofacial defects,^[7]^ while their application is constrained by donor-site morbidity and a scarcity of grafted tissue.^[8]^ Fortunately, allografts or xenografts can solve the aforementioned issues, whereas the effectiveness of these procedures for bone regeneration was not as high as that of autografts.^[9]^ Similarly, biocompatible artificial materials can be used to restore nerve injuries and repair bone deformities in the oral and maxillofacial region, but there are disadvantages such as secondary damage, unsightliness, long cycle time, high expense, and a strong sensation of foreign bodies.^[10]^ Moreover, the damage of oral and maxillofacial blood vessels, nerves, teeth and articular cartilage, and other tissues seriously jeopardizes the physical and mental health of patients.^[11]^ Therefore, tissue regeneration of the oral and maxillofacial region is still an important challenge faced by dentistry at present.

In recent years, cell therapy based on stem cells is one of the important strategies for tissue regeneration and repair.^[12,13]^ Stem cells have become important “seed cells” in tissue engineering due to their self-renewal and multidirectional differentiation potential, and have a broad application prospect in the treatment of bone diseases, immune disorder diseases, neurodegenerative diseases, and malignant tumors.^[14–17]^ Stem cells can be isolated from a variety of human tissues, such as placenta, bone marrow, umbilical cord, adipose, dental pulp,^[18]^ etc. However, there are some problems with stem cell therapy, such as body immune rejection, mutation of stem cell genetic material, and in vitro expansion not easy to preserve.^[19]^ Increasing evidence has been found that stem cells can release a large number of extracellular nanoparticles to participate in the process of tissue injury and repair, and exosomes have attracted much attention as one of them.^[20]^ Exosomes are micro-vesicles released by eukaryotic cells that include a variety of proteins, lipids, and genetic material, which are involved in both physiological and pathological processes, such as intercellular communication and genetic material transfer.^[21]^ In particular, stem cell-derived exosomes have the advantages of high proliferation capacity and low immunogenicity.^[22]^ Huang et al^[23]^ reported that human bone marrow mesenchymal stem cell (MSC)-derived exosomes promoted bone regeneration. Numerous studies have proved that stem cell-derived exosomes promoted bone or nerve regeneration.^[24–26]^ Among them, dental pulp stem cell (DPSC) and periodontal ligament stem cell (PDLSC)-derived exosomes have been reported to act as a new therapeutic strategy for use in bone regeneration.^[27,28]^ Therefore, MSC-derived exosomes may serve as a promising non-cell source for regenerative medicine and tissue engineering in the oral and maxillofacial region.

Herein, we summarize the biological functions of stem cell-derived exosomes. Moreover, we discuss the functional roles of stem cell-derived exosomes in bone and nerve tissue regeneration, as well as their potential mechanisms. This review aims to provide new insights into the application of stem cell-derived exosomes for the treatment of oral and maxillofacial diseases.

## 2. Materials and methods

### 2.1. Searching strategy

This review article was conducted using electronic databases such as PubMed and Web of Science. All published data till the year 2025 have been taken into consideration. The following search keywords were used in the search of materials for this study: “oral and maxillofacial diseases,” “periodontal regeneration,” “stem cells,” “exosome,” “dental pulp,” “periodontium,” “jawbone,” “temporomandibular joint,” “maxillofacial soft tissue,” “nerve repair,” “inflammation,” “wound healing,” “angiogenesis,” “tissue regeneration,” and other similar keywords in combination with words such as stem cell, exosomes, clinical trials, and human health. All articles addressing these principal keywords were considered when available in the English language, and in peer-reviewed journals, whether published as review or research articles. Papers were reviewed in their entirety if their abstract mentioned that the article presented any potential relevance to the inclusion criteria. Articles were excluded based on title, abstract, or full text because of their lack of pertinence to the issue concerned. Articles were excluded if they were letters, comments, or not available for access to full article.

### 2.2. Research ethics

Ethical approval was not required, as the data used in this article were downloaded from the public databases and did not involve interaction with human participants.

## 3. Overview of exosomes

### 3.1. Origin, secretion mechanism, and structure of exosomes

**Figure 1.**
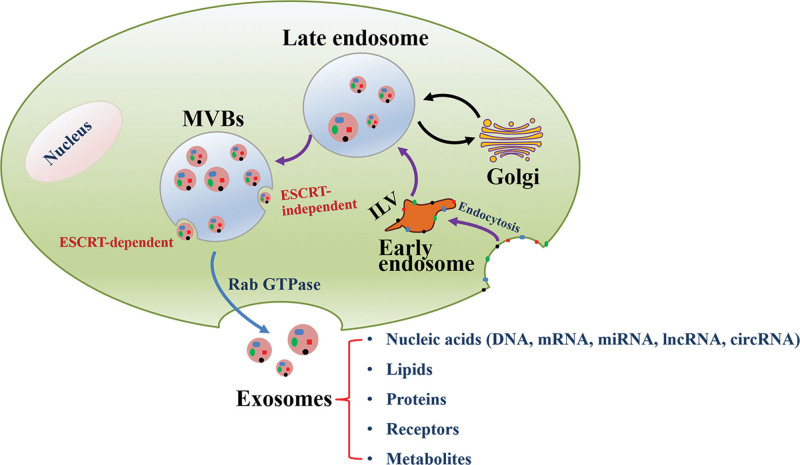
Origin of exosomes and distribution of different exosomal common cargo molecules. ESCRT = endosomal sorting complexes required for transport, ILV = intraluminal vesicle, MVBs = multivesicular bodies.

Exosomes belong to a category of extracellular vesicles with a diameter of 30 to 150 nm,^[29]^ which are secreted by all cell types and have been found in various body fluids. Exosomes originate from late endosomes and are generated by the double invagination of the plasma membrane and the formation of intracellular multivesicular bodies.^[30,31]^ Multivesicular bodies would fuse with the cellar plasma membrane and proceed exocytosis to release exosomes through endosomal sorting complexes required for transport-dependent^[32]^ and endosomal sorting complexes required for transport-independent mechanisms^[33]^ (Fig. 1). Consistent with the biogenesis, abundant molecular information of exosomes reflects closely the parent cells.^[34,35]^ Exosomes are surrounded by the lipid bilayer membrane, which is composed of proteins and lipids, while most exosome proteins are tetraspanins.^[36,37]^ Moreover, multiple types of RNA have been identified in exosomes, including mRNA and non-coding RNAs such as microRNA, long non-coding RNA (lncRNA), and circular RNA.^[38,39]^ Previous studies have proved that different preconditioning of cells, such as hypoxia, ischemia, and lipopolysaccharide, can cause them to release exosomes containing different “cargoes.”^[40,41]^ Recently, several exosomal marker proteins were identified, such as tetraspanin families (CD63, CD9, CD81), heat shock proteins (HSP70, HSP90), and tumor susceptibility gene 101 protein.^[21]^ Electron microscopy observed that the morphology of exosomal vesicles was elliptical or cup-shaped particles.^[42]^

### 3.2. Biological functions of exosomes

Exosomes have been used in preclinical models for antitumor therapy since 1990.^[43]^ In recent years, exosomes treatment contributed to periodontal regeneration by inhibiting bone resorption and initiating bone repair, as well as the inhibition of inflammatory responses.^[44,45]^ Chew et al^[46]^ showed that bone marrow MSC-derived exosomes could promote periodontal regeneration through the activation of the protein kinase B/extracellular regulated protein kinase (ERK) pathway to enhance the migration and proliferation of PDLSCs. Wang et al^[47]^ showed that gingival MSCs-derived exosomes could promote the polarization of M1-type macrophages to M2-type and inhibit the inflammatory response, which provided a possible approach for the treatment of periodontitis. Compared with conventional therapies, exosomes have gradually replaced cell therapy as an effective alternative for treating periodontitis and improving alveolar bone resorption because of their advantages such as small size, low immunogenicity, high stability, ability to cross the blood–brain barrier, ease of storage, and no need for additional culture expansion.^[48,49]^ In addition, exosomes, as mediators of intercellular information transfer, carry disease-specific biomarkers, such as mutated genes, mRNAs, non-coding RNAs, etc, which have great potential for disease diagnosis and enable earlier, sensitive, and specific disease diagnosis.^[50,51]^

## 4. Stem cell-derived exosomes and oral and maxillofacial tissue regeneration

### 4.1. Exosomes and pulp tissue regeneration

**Figure 2.**
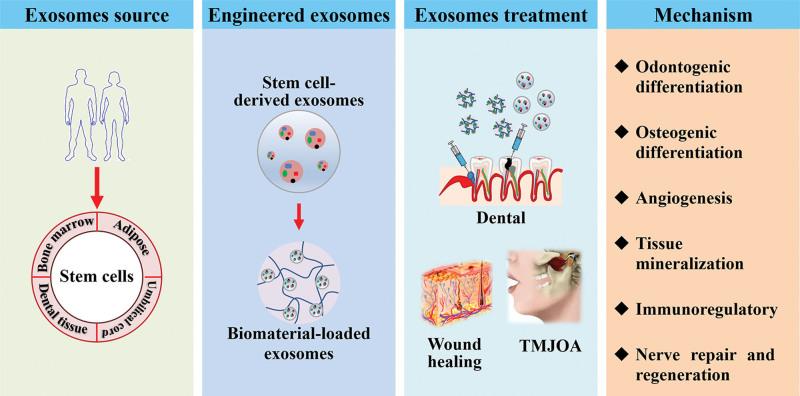
Functional role and mechanism of stem cell-derived exosomes in periodontal regeneration. TMJOA = temporomandibular joint osteoarthritis.

Previous studies have found that stem cell-derived exosomes contributed to the dentin–pulp complex and pulp tissue regeneration by inducing stem cell differentiation into pulp cells and/or dentin-forming cells, axonal regeneration of peripheral nerves, and migration of vascular endothelial cells (Table 1 and Fig. 2).^[88,89]^ Among them, angiogenesis is the basis for the regeneration of the pulp–dentin complex, and adequate blood supply is necessary for tissue regeneration.^[90,91]^ Numerous studies have demonstrated that DPSC-derived exosomes promoted human umbilical vein endothelial cell proliferation, proangiogenic factor expression, and tube formation.^[92–94]^ Guo et al^[57]^ found that exosomes derived from human DPSCs aggregated initiate functional tooth regeneration by upregulating the odontogenic and angiogenic ability of human DPSCs. Wu et al^[95]^ reported that stem cells from exfoliated deciduous teeth aggregate-derived exosomes improved pulp tissue regeneration and angiogenesis. Another study showed that exosomes derived from DPSCs stably overexpressing hypoxia-inducible factor-1α promoted endothelial cell tube formation and angiogenesis in vivo.^[96]^ Moreover, dentin matrix protein-1, dentin salivary protein, and dentin phosphoprotein play important roles in dentin formation and mineralization.^[97]^ For instance, apical papilla stem cell-derived exosomes were endocytosed by bone marrow MSCs and promoted dentin–pulp complex regeneration through inducing specific dentinogenesis.^[64]^ Human DPSC-derived exosome treatment resulted in dentin bridge formation in a rat pulp-capping model by upregulation of dentinogenic gene (dentin matrix protein-1, dentin salivary protein, and dentin phosphoprotein) expression and mineralization.^[98]^ Zhang et al^[60]^ showed that exosomes derived from Hertwig epithelial root sheath cells promote the regeneration of dentin–pulp tissue by inducing odontogenic differentiation of dental papilla cells. What’s more, DPSC-derived exosomes contributed to sciatic nerve regeneration^[81]^ and repair.^[82]^ Similarly, exosomes derived from lipopolysaccharide-preconditioned DPSCs promoted the proliferation, migration, and odontogenic differentiation of Schwann cells.^[99]^ These results indicated that stem cell-derived exosomes exhibited a promotion effect on pulp tissue regeneration, especially dental stem cells.

**Table 1:** Summary of stem cell-derived exosomes in the treatment of oral and maxillofacial diseases.

Stem cell source	Efficacy and mechanism	References
Bone repair and regeneration		
BMMSCs	♢Bone loss↓ ♢Proliferation, migration, and osteogenic differentiation of BMSCs↑ ♢miR-26a↑and DP7-C and mTOR pathway↓	^[52]^
BMMSCs	♢Alveolar bone loss, inflammatory infiltration, and collagen destruction↓ ♢M2 macrophages↑and OPG-RANKL-RANK pathway↓	^[53]^
DPSCs	♢Bone tissue regeneration↑ ♢Upregulated miRNAs (miR-29c-5p, miR-378a-5p, miR-10b-5p, and miR-9-3p) associated with osteogenesis ♢Down-regulated anti-osteogenic miRNA (miR-31-3p, miR-221-3p, miR-183-5p, and miR-503-5p)	^[28]^
DFSCs	♢The proliferation and migration of extruded nanovesicles↑ ♢Periodontal tissue regeneration↑	^[54]^
DPSCs	♢The expression of RUNX2, ALP, and OCN↑ ♢Osteogenic differentiation capability of JB-MSCs↑ ♢New bone density↑	^[55]^
DPSCs	♢The migration of both dental pulp cells and osteoblastic cells↑ ♢The proliferation of dental pulp cells↑ ♢Alveolar bone resorption and osteoclast formation↓	^[56]^
DPSCs	♢Odontogenic and angiogenic ability of DPSCs↑ ♢Regeneration of functional teeth↑	^[57]^
DPSCs	♢Odontogenic differentiation of DPSCs↑ ♢Regeneration of dental pulp-like tissue and p38 MAPK pathway↑	^[58]^
GMSCs	♢Periodontal bone resorption, number of TRAP-positive osteoclasts, and Wnt pathway↓	^[59]^
HERSCs	♢The proliferation and migration of dental papilla cells↑ ♢Odontogenic differentiation of dental papilla cells↑ ♢Tube formation and neural differentiation↑ ♢Dental pulp tissue regeneration and Wnt/β-catenin pathway↑	^[60]^
PDLSCs	♢Alveolar bone destruction and number of osteoclasts↓ ♢miR-31-5p↑and eNOS↓	^[61]^
PDLSCs	♢Osteogenic differentiation of PDLSCs and bone formation↑ ♢Wnt pathway↓	^[44]^
PDLSCs	♢The proliferation and osteogenic differentiation of BMSCs↑ ♢New bone formation in alveolar bone defects↑	^[62]^
PDLSCs	♢The proliferation, migration and osteogenic differentiation of hFOB1.19 cell, PI3K/AKT and MEK/ERK pathways↑	^[63]^
PDLSCs	♢Regeneration of critical-size periodontal defects↑ ♢The proliferation and migration of periodontal ligament↑ ♢Akt/ERK pathway↑	^[46]^
SCAP	♢Dental pulp-like tissues and newly formed dentin↑ ♢The expression of dentin sialophosphoprotein and mineralized nodule formation in BMMSCs↑	^[64]^
SHED	♢The expression of angiogenesis-related genes (KDR, SDF-1, and FGF2), osteogenesis-related genes (COL1, RUNX2, and OPN)↑ ♢Neovascularization, new bone formation, and AMPK pathway↑	^[65]^
UCMSCs	♢HUVEC proliferation, migration, and tube formation↑ ♢Alveolar bone defect repair, angiogenesis, and osteogenesis↑	^[66]^
Periodontitis		
BMMSCs	♢The levels of TNF-α, IL-1β, IL-6, and IL-8↓ ♢p38/ELK1 pathway↑	^[67]^
DPSCs	♢The proliferation, migration, and osteogenesis of PDLSCs↑ ♢M2 macrophages and healing of the periodontal epithelium↑ ♢The expression of IL-1β and TNF-α and JAK2/STAT3 pathway↓	^[68]^
DPSCs	♢Levels of IL-23, IL-1α, TNF-α, IL-12, IL-1β, IL-27, and IL-17↓ ♢miR-1246 and M2 macrophages↑ ♢NF-κB and p38 MAPK pathway↓	^[69]^
GMSCs	♢The expression of IL-1β, TNF-α↓and IL-10↑ ♢NF-κB pathway and Wnt5a↓	^[70]^
PDLSCs	♢Levels of TNF-α, IL-1β, and IL-6, and percentage of Th17 cells↓ ♢miR-205-5p↑and XBP1↓	^[71]^
PDLSCs	♢miR-143-3p↑ ♢M1 macrophage polarization and PI3K/Akt/NF-κB pathway↓	^[72]^
SHED	♢The proliferation and osteogenic differentiation of PDLSCs↑ ♢The apoptosis and inflammatory responses in PDLSCs↓ ♢miR-92a-3p↑and PI3K/AKT pathway↓	^[73]^
Wound healing		
ADMSCs	♢Levels of TNF-α and IL-1β↓ ♢Primary gingival wound healing and bone regeneration↑ ♢The migration and collagen human gingival fibroblasts↓	^[74]^
BMMSCs	♢Wound closure and bone volume/tissue volume↑ ♢The expression of IL-6, IL-8, MMP1/3↓ ♢Proliferation and migration of fibroblasts↑ ♢Bisphosphonate-related osteonecrosis of the jaw↓	^[75]^
Temporomandibular joint osteoarthritis		
Human embryonic MSCs	♢Pain, temporomandibular joint degeneration, and inflammation↓ ♢Subchondral bone deterioration and matrix degradation↓ ♢IL-β^+^ and iNOS^+^ cells↓ ♢Cell proliferation and Akt/ERK/AMPK pathway↑	^[76]^
SHED	♢The expression of IL-6, IL-8, MMP1/3/9/13↓ ♢miR-100-5p↑and mTOR↓	^[77]^
Urine-derived stem cells	♢The structural integrity and smoothness of the compromised condylar cartilage surface↑ ♢Osteoclastogenic activity and inflammation↓	^[78]^
Peripheral nerve regeneration		
ADMSCs	♢Sensory and motor function recovery↑ ♢Schwann cell proliferation and migration↑ ♢CSF-1/CSF-1R pathway↓	^[79]^
BMMSCs	♢The proliferation and migration of Schwann cells↑ ♢Facial nerve repair and regeneration↑ ♢circRNA_Nkd2↑and miR-214-3p↓	^[80]^
DPSCs	♢The proliferation, migration, and secretion of neurotrophic factors by Schwann cells↑ ♢Neurite elongation and axon regeneration↑	^[81]^
DPSCs	♢Schwann cell autophagy and p53 expression↓ ♢Regeneration of the myelin sheath and miR-122-5p↑	^[82]^
Endometrial stem cells	♢Nerve regeneration and formation of regenerated nerve fibers↑ ♢Newly blood vessels↑	^[83]^
GMSCs	♢Schwann cell proliferation and axon growth↑ ♢The number and diameter of nerve fibers and myelin formation↑	^[84]^
Muscle stem cells	♢Schwann cell ferroptosis↓and cell proliferation↑ ♢Keap1/Nrf2/HO-1 pathway↑	^[85]^
Pluripotent stem cells	♢The proliferation of Schwann cells↑ ♢The process of axonal regeneration and remyelination↑	^[86]^
UCMSCs	♢Peripheral nerve regeneration and restoration of motor function↑ ♢CD31-positive endothelial cells↑ ♢Axon regeneration and distal nerve reconnection↑	^[87]^

ADMSCs = adipose-derived mesenchymal stem cells, Akt = protein kinase B, ALP = alkaline phosphatase, AMPK = AMP-activated protein kinase, BMMSCs = bone marrow mesenchymal stem cells, circRNA = circular RNA, CSF-1R = colony-stimulating factor-1 receptor, DFSCs = dental follicle stem cells, DPSCs = dental pulp stem cells, GMSCs = gingival tissue-derived mesenchymal stem cells, HERSCs = Hertwig epithelial root sheath cells, IL = interleukin, JB-MSCs = jawbone marrow-derived mesenchymal stem cells, MEK = mitogen-activated protein kinase, miRNA = microRNA, MMP = matrix metalloproteinase, OCN = osteocalcin, PDLSCs = periodontal ligament stem cells, RUNX2 = runt-related transcription factor, SCAP = stem cells from apical papilla, SHED = stem cells from human exfoliated deciduous teeth, TRAP = tartrate-resistant acid phosphatase, UCMSCs = umbilical cord mesenchymal stem cells.

### 4.2. Exosomes and periodontal regeneration

Periodontitis is a complicated chronic inflammatory oral disease that seriously threatens oral health and quality of life.^[100]^ In recent years, as a novel intercellular communication medium, the role of exosomes in the pathogenesis, diagnosis, and treatment of periodontitis has gradually received widespread attention (Table 1).^[49]^ For example, DPSC-derived exosomes inhibited periodontitis and stimulated tissue regeneration by promoting the proliferation, migration, and osteogenesis of PDLSCs.^[68]^ Similarly, PDLSC-derived exosome treatment resulted in improved inflammatory bone loss in rat models of periodontitis.^[44]^ Chew et al^[46]^ also showed that PDLSC-derived exosomes promoted periodontal regeneration by enhancing periodontal ligament cell proliferation and migration. Deng et al^[101]^ reported that MSC-derived exosomes exhibited robust immunomodulatory effects against periodontitis by promoting periodontal regeneration. Zhang et al^[102]^ showed that exosomes derived from 3D-cultured DPSCs suppressed periodontitis by restoring the Th17 cell/Treg balance. Moreover, MSC-derived exosomes displayed immunomodulatory and anti-inflammatory effects on periodontitis.^[103]^ For example, MSC-derived exosomes reduced the number of osteoblasts and fibroblasts, as well as suppressed inflammation, thereby contributing to angiogenesis and bone regeneration.^[75]^ Liu et al^[53]^ found that bone marrow MSC-derived exosomes promoted periodontal regeneration by increasing the ratio of macrophage type 2. Nakao et al^[59]^ showed that exosomes secreted from tumor necrosis factor-alpha-treated human gingiva tissues-derived MSCs suppressed periodontal bone loss by upregulating M2 macrophage polarization. Similarly, exosomes secreted from gingival MSCs^[47]^ and PDLSCs^[104]^ ameliorated periodontitis by inhibiting M1 and promoting M2 macrophage polarization. The switch between different phenotypes of macrophages is closely related to the tissue destruction caused by periodontitis, and various cytokines and inflammatory mediators secreted from macrophages are involved in the destruction and repair of periodontal tissue.^[105,106]^ As expected, reparative M2-like macrophage-derived exosomes prevented periodontitis progression by reducing bone loss and inflammation.^[107]^ Of note, M2 macrophage-derived exosomes facilitated the osteogenic differentiation of human PDLSCs.^[108]^ Taken together, exosomes derived from stem cells were an effective therapeutic strategy for the treatment of periodontitis.

## 5. Stem cell-derived exosomes and oral and maxillofacial bone tissue regeneration

### 5.1. Exosomes and jawbone regeneration

Previous studies have proved that stem cell-derived exosomes have been shown to promote bone regeneration by regulating osteogenic differentiation (Table 1).^[109,110]^ For example, DPSC-derived exosomes alleviated jawbone defects by enhancing the expression of osteogenic genes (such as ALP, RUNX2, and OCN) and promoting the osteogenic differentiation of jawbone marrow-derived MSCs.^[55]^ Exosomes from human adipose-derived MSCs stabilized the bone graft environment, ensured blood supply, and accelerated cartilage and bone regeneration.^[111,112]^ A recent study showed that human umbilical cord MSC-derived exosomes promoted jawbone regeneration by increasing the expression of osteogenic genes.^[113]^ Other studies have proved that MSC-derived exosomes promoted the restoration of jawbone defects by upregulating bone-inducible non-coding RNAs, such as miR-381^[114]^ and lncRNA MALAT1.^[115]^ Moreover, exosomes secreted by adipose-derived MSC transfected with BMP2 and VEGFA accelerated bone regeneration by promoting angiogenesis and osteogenic differentiation.^[116]^ Lian et al^[117]^ demonstrated that nerve growth factor-preconditioned MSC-derived exosomes contributed to innervated bone regeneration. The above results indicated that stem cell-derived exosomes promoted jawbone regeneration may be related to their regulation of osteogenic differentiation, revascularization, and neural innervation.

### 5.2. Exosomes and cartilage regeneration

Recently, stem cell-derived exosomes have been shown to promote cartilage repair and regeneration (Table 1).^[118,119]^ For instance, exosomes from urine-derived stem cells ameliorated temporomandibular joint osteoarthritis (TMJOA) by promoting cartilage regeneration.^[78]^ Zhang et al^[76]^ reported that intra-articular injection of human embryonic stem cell-derived exosomes alleviated pain and TMJOA by reducing inflammation and improving matrix homeostasis, and activating the protein kinase B/ERK/AMP-activated protein kinase pathway. Similarly, stem cell-derived exosomes were also used to treat osteoarthritis by maintaining chondrocyte^[120]^ and mitochondrial^[121]^ function, suppressing inflammation,^[122]^ and regulating the balance of synthesis and catabolism of extracellular matrix.^[123]^ Moreover, chondrocyte-derived exosomes facilitated TMJOA by promoting cartilage calcification.^[124]^ Taken together, stem cell-derived exosomes were an effective therapeutic strategy for cartilage regeneration, and suppression of chondrocyte-derived exosomes was a new way to prevent TMJOA.

## 6. Stem cell-derived exosomes and oral and maxillofacial wound healing

Non-healing maxillofacial skin wounds may aggravate the patient’s condition, affect the patient’s quality of life, and increase the patient’s psychological burden. Stem cell-derived exosomes have been shown to accelerate oral and maxillofacial wound healing (Table 1).^[125,126]^ For example, jawbone marrow MSC-derived exosomes accelerated wound healing by inducing macrophage M2 polarization.^[127]^ He et al^[128]^ showed that exosomes from adipose-derived MSCs promoted wound healing by upregulating lncRNA MALAT1 and activation of the Wnt/β-catenin pathway. Similarly, bone marrow MSC-derived exosomal miR-542-3p promoted wound repair and regeneration by regulating skin fibroblasts and dermal microvascular endothelial cells.^[129]^ Other studies found that stem cell-derived exosomes facilitated wound healing by promoting angiogenesis and skin cell proliferation and reepithelization.^[130,131]^ Moreover, stem cell-derived exosomes inhibited scar formation by modulating collagen remodeling in the process of wound healing.^[132,133]^ For example, exosomes from adipose-derived MSCs alleviated hypertrophic scar fibrosis by reducing the deposition of collagen and pro-fibrotic protein levels.^[134]^ Currently, biomaterials loaded with exosomes have attracted wide attention as an emerging therapeutic strategy in the field of tissue engineering and regenerative medicine, and are considered as a promising means for the treatment of wound healing with good biocompatibility.^[135,136]^ Taken together, stem cell-derived exosomes promoted oral and maxillofacial wound healing by inhibiting inflammation, promoting fibroblast proliferation and angiogenesis, and reducing scar formation.

## 7. Stem cell-derived exosomes and oral and maxillofacial peripheral nerve regeneration

Increasing evidence has demonstrated that stem cell-derived exosomes promoted peripheral nerve regeneration in experimental models (Table 1).^[137,138]^ Chai et al^[82]^ reported that DPSC-derived exosomes attenuated sciatic nerve injury by promoting myelin sheath regeneration and inhibiting Schwann cell autophagy. Another study showed that DPSC-derived exosomes promoted sciatic nerve regeneration by promoting the proliferation and migration of Schwann cells, as well as inducing neurotrophic factor secretion.^[81]^ Recently, engineered exosomes served as a useful and novel therapeutic strategy for the treatment of peripheral nerve injury. For example, electroconductive hydrogel-loaded bone marrow MSC-derived exosomes promoted Schwann cell proliferation and migration and myelinated axonal regeneration by activating the mitogen-activated protein kinase/ERK pathway.^[139]^ Similarly, biodegradable chitin conduits combined with exosomes from human gingiva-derived MSCs facilitated sciatic nerve regeneration and Schwann cell proliferation.^[84]^ Wang et al^[140]^ proved that neuroprotective peptide PACAP38-loaded exosomes promoted axon regeneration and improved nerve function after injury. Collectively, stem cell-derived exosomes may act as a promising cell-free therapeutic approach for the treatment of oral and maxillofacial peripheral nerve injury.

## 8. Clinical trials of stem cell-derived exosomes for oral and maxillofacial tissue regeneration

Exosomes derived from odontogenic stem cells have the advantage of good biocompatibility and low immunogenicity, and serve as new therapeutic drugs for tissue repair and regeneration.^[88]^ According to the official database of the National Institutes of Health, more than 100 clinical studies of stem cell-derived exosomes were used for the prevention and treatment of various diseases. Meanwhile, the clinical trials of stem cells and their exosomes in oral and maxillofacial tissue regeneration are gradually increasing, which we have summarized in Table 2. Currently, several clinical studies have reported that stem cell-derived exosomes are a safe, effective, and feasible therapeutic method for tissue regeneration.^[141,142]^ An early phase I clinical trial is ongoing to evaluate the safety, tolerability, and efficacy of adipose MSC-derived exosomes in the treatment of periodontitis (NCT04270006). A pilot study showed that Wharton jelly MSC-derived exosomes improved peripheral nerve damage and promoted axonal regeneration.^[143]^ Another clinical trial has completed the safety and tolerability study of adipose MSC-derived exosomes for wound healing (NCT05475418). These clinical trials suggested that oral and maxillofacial wounds can be improved and cured by exosomes secreted by stem cells. However, most of the clinical trials based on stem cell-derived exosomes for the treatment of oral and maxillofacial tissue regeneration are in recruitment status, that is, phase I or phase II studies, which suggests that there is still a long way to go for the application of exosomes in clinical applications. While the field is advancing, with some therapies already approved for clinical use, significant challenges remain. These include standardizing methods for obtaining high-purity exosomes, scaling up production, and fully elucidating their complex mechanisms of action. Future efforts are focused on the engineering of exosomes for improved targeting and loading of specific therapeutic molecules, and their integration with sophisticated 3D-printed and layered scaffold designs to achieve the synchronized regeneration of complex tissue structures like the periodontium.

**Table 2:** Clinical trials of stem cells and their exosomes in periodontal regeneration.

Source	Year	Sponsor	Status	Clinical trial ID
PDLSCs	2011	Air Force Military Medical University, China	Unknown	NCT01357785
BMMSCs	2014	Aristotle University Of Thessaloniki, Greece	Completed	NCT02449005
DPSCs	2014	Songlin Wang	Unknown	NCT02523651
UCMSCs	2016	Universidad de los Andes, Chile	Completed	NCT03102879
DPSCs	2016	University of Turin, Italy	Completed	NCT03386877
BMMSCs	2018	Instituto Venezolano de Investigaciones Cientificas, Venezuela	Enrolling by invitation	NCT05975892
BMMSCs	2019	Universidad Central de Venezuela, Venezuela	Completed	NCT04545307
ADMSCs-Exo	2020	Beni-Suef University, Egypt	Unknown	NCT04270006
BMMSCs	2020	University of Bergen, Norway	Unknown	NCT04297813
ADMSCs-Exo	2022	Shanghai Ninth People’s Hospital Affiliated to Shanghai Jiao Tong University, China	Completed	NCT05475418
ADMSCs-Exo	2024	Pontificia Universidade Católica do Rio Grande do Sul, Brazil	Not yet recruiting	NCT04998058

ADMSCs = adipose-derived mesenchymal stem cells, BMMSCs = bone marrow mesenchymal stem cells, DPSCs = dental pulp stem cells, PDLSCs = periodontal ligament stem cells, UCMSCs = umbilical cord mesenchymal stem cells.

## 9. Conclusion and perspective

Over the past decades, stem cell-derived exosomes have received increasing attention as a major focus of regenerative medicine research. Increasing evidence has proved that stem cell-derived exosomes have many advantages: abundant sources, easy to obtain and store; good biocompatibility and stability; easy to pass the blood–cerebrospinal fluid barrier, and can be used as a natural molecular carrier material for transporting drugs; avoid the risk of stem cell transplantation therapy. Meanwhile, stem cell-derived exosomes show tremendous therapeutic potential for regenerative dentistry, especially exosomes from odontogenic stem cells. Mechanistically, stem cell-derived exosomes alleviated oral and maxillofacial diseases by promoting tissue regeneration, osteogenesis, odontogenesis, angiogenesis, axonal regeneration, and suppressing inflammation. Moreover, engineered exosomes also exhibited promotion effects on dental tissue regeneration. However, the successful clinical translation of exosome-based therapies still faces several critical challenges. Firstly, a standard for MSC-derived exosome acquisition, such as the isolation, purification, optimization, quality control, and preservation of exosomes. Secondly, the exact molecular mechanisms of exosome communication with target cells still need to be further studied, and the secretion mechanism of stem cell-derived exosomes remains unknown. Thirdly, injection methods, dosage, and treatment course of stem cell-derived exosomes. Fourthly, the safety and efficacy of stem cell-derived exosomes in clinics need to be further confirmed. Overall, solving these problems not only promotes in-depth mechanism research, but also has important significance for clinical translational application of stem cell-derived exosomes for oral and maxillofacial diseases.

## Author contributions

**Conceptualization:** Qianfen Dai.

**Data curation:** Qianlei Dai.

**Investigation:** Qianlei Dai, Qianfen Dai.

**Methodology:** Qianlei Dai, Qianfen Dai.

**Resources:** Qianlei Dai.

**Writing – original draft:** Qianlei Dai.

**Writing – review & editing:** Qianfen Dai.
